# The Phosphorylation of CCR6 on Distinct Ser/Thr Residues in the Carboxyl Terminus Differentially Regulates Biological Function

**DOI:** 10.3389/fimmu.2018.00415

**Published:** 2018-03-02

**Authors:** Mei-Yi Lu, Syuan-Shao Lu, Shiann-Luen Chang, Fang Liao

**Affiliations:** ^1^Institute of Biomedical Sciences, Academia Sinica, Taipei, Taiwan

**Keywords:** CCR6, G protein-coupled receptor, mutagenesis, receptor phosphorylation, β-arrestins

## Abstract

CCR6 is a G protein-coupled receptor (GPCR) that recognizes a single chemokine ligand, CCL20 and is primarily expressed by leukocytes. Upon ligand binding, CCR6 activates Gαi heterotrimeric G proteins to induce various potential cellular outcomes through context-specific cell signaling. It is well known that differential phosphorylation of Ser and Thr residues in the C-terminal domains or intracellular loops of GPCRs can generate barcodes that regulate GPCR function by regulating the recruitment of β-arrestins. In this study, we demonstrate that ligand binding to CCR6 induces receptor phosphorylation at Ser/Thr residues in the C-terminal tail, rather than intracellular loops. Using mutagenesis experiments, we determined that distinct clusters of Ser/Thr residues in the C-terminal domain differentially regulate CCL20-induced signaling and cellular response. Substituting the Thr360/Ser361/Thr363 cluster or the Ser370/Ser371 cluster with Ala residues modulated cellular response upon CCL20 stimulation. Notably, receptor internalization, chemotaxis, F-actin distribution, transient ERK1/2 activation, and β-arrestin 2 recruitment were oppositely affected by mutating the two clusters, suggesting that phosphorylation of CCR6 C-terminal Ser/Thr residues directs the cell signaling response upon receptor activation. Moreover, activated CCR6 weakly recruited β-arrestin 1 in comparison with β-arrestin 2, and the two arrestin proteins seemed to play overlapping but distinct roles in mediating CCL20/CCR6-induced cellular responses. Taken together, the effects of site-specific Ser/Thr phosphorylation on CCR6 demonstrate the existence of barcodes on the protein that dictate the activation of different cell signaling profiles and lead to distinct biological outcomes.

## Introduction

CCR6 is a chemokine receptor, which belongs to the G protein-coupled receptor (GPCR) superfamily. The protein is mainly expressed on immature dendritic cells, B cells, and effector/memory T cells (1–4). Of particular interest, CCR6 has been shown to be an exclusive chemokine receptor on Th17 cells (5–8) and group 3 innate lymphoid/ILC3 cells (9, 10), suggesting a role for the receptor in a number of inflammatory and autoimmune diseases (6, 11, 12), especially those related to mucosal immunity (13–15). CCL20 is the only known chemokine ligand for CCR6 (2, 4, 16). Upon binding to CCL20, CCR6 on leukocytes initiates signal transduction *via* activation of the Gαi family of heterotrimeric G proteins (16, 17). Furthermore, like most classical chemokine receptors, the downstream signaling of CCR6 has been shown to involve activation of calcium mobilization, PLC-β, phosphatidylinositol 3-kinase/Akt, ERK1/2 phosphorylation, and actin polymerization (2, 3, 17–21).

It is well known that upon GPCR activation, certain Ser and Thr residues within intracellular loops or the C-terminal domains of the receptors are phosphorylated by G protein-activated receptor kinases (GRKs) or secondary messenger kinases, such as PKA or PKC. These phosphorylated intracellular domains of GPCRs then recruit β-arrestins (β-arrestin 1 and/or β-arrestin 2) to uncouple the receptor from heterotrimeric G proteins and facilitate receptor internalization and desensitization (22–24). Although β-arrestins play a well-defined role in terminating G-protein-mediated signaling, these multifunctional adaptors have also been reported to function as scaffolds for many signaling molecules, including mitogen-activated protein kinase (MAPK), c-Src, and Akt, among others (22). The site-specific phosphorylation of C-terminal Ser/Thr residues in GPCRs often selects for a specific spatiotemporal pattern of adaptor and signaling molecule associations that exist within the wide-ranging repertoire of an individual GPCR. Thus, it is important to delineate the effects of C-terminal phosphorylation sites on the biological consequences of GPCR activation (25–28).

Similar to other GPCRs, the C-terminal domains of many chemokine receptors contain Ser/Thr residues that are phosphorylated upon ligand stimulation and increase binding affinity for arrestin proteins (29, 30). Although C-terminal phosphorylation of chemokine receptors is critical for chemokine-induced internalization, the effects of phosphorylation on receptor-mediated intracellular signaling and biological function vary with receptor and cell types. Previous studies have provided details regarding the biological functions of phosphorylation on the C-terminal tail for CXCR1, CXCR2, CXCR3, CXCR4, CCR5, and CCR7 (31–36). However, the role of the C-terminal tail in CCR6-mediated signaling has not yet been reported.

To better understand how specific Ser/Thr residues in the C-terminal domain of CCR6 regulate protein function, we generated a series of CCR6-Jurkat stable cell lines that express CCR6 with alanine mutations at certain Ser/Thr residues. Using these constructs, we demonstrated that different C-terminal Ser/Thr phosphorylation sites in CCR6 are distinctly involved in the regulation of CCR6-activation-mediated effects, including receptor internalization, cell migration, F-actin distribution, and ERK1/2 activation. In addition, we showed that the activated CCR6 recruits both β-arrestin 1 and β-arrestin 2, but with different affinities. Notably, β-arrestin 1 and β-arrestin 2 appear to play discrete roles in modulating CCR6 activity. Thus, phosphorylation of individual C-terminal Ser/Thr residues and β-arrestin recruitment combine to determine the landscape of CCR6 functionality, indicating that a phospho-barcode dictates distinct signaling outcomes and directs the biological function of the receptor.

## Materials and Methods

### Cell Culture and Transfection

Jurkat cells (human T cell lymphoblast-like cell line) were transfected with the plasmid, pCIneo (Promega, Madison, WI, USA), encoding human HA-tagged CCR6-wild-type (WT) or HA-tagged CCR6 mutants. Multiple clones were established by FACS Aria cell sorter (Becton Dickinson, San Joes, CA, USA). CCR6-expressing Jurkat cells were maintained in RPMI 1640 medium supplemented with 10% FBS, 2 mM l-glutamine, NEAA, 1 mM sodium pyruvate, 5.5 µM β-mercaptoethanol and 1 mg/ml G418 (Life Technologies, Grand Island, NY, USA). β-Arrestin 2-GFP-expressing U2OS cells were maintained in minimum essential medium supplemented with 10% FBS, 2 mM l-glutamine and 0.4 mg/ml G418. HEK293T and U2OS cells were maintained in Dulbecco’s modified Eagle’s medium supplemented with 10% FBS and 2 mM l-glutamine. Jurkat cells were transfected with 500 nM non-targeting control siRNA (Dharmacon, Lafayette, CO, USA), β-arrestin 1 siRNA (target sequence: CTCGACGTTCTGCAAGGTCTA) or β-arrestin 2 siRNA (target sequence: CTCGAACAAGATGACCAGGTA) (Qiagen, Germantown, MD, USA) by electroporation using a BTX Pulse Generator ECM 630 system (Genetronics, Inc., San Diego, CA, USA). Electroporation conditions were 260 mV, 725 mΩ, and 1, 050 μF. U2OS and HEK293T cells were transfected with plasmids encoding HA-tagged CCR6-WT or HA-tagged CCR6 mutants using Lipofectamine 2000 (Invitrogen, Carlsbad, CA, USA) according to manufacturer’s instruction.

### Constructs

FLAG-tagged rat β-arrestin 1 was obtained as a gift from Dr. Lee Yuan Liu-Chen (Temple University, Philadelphia, PA, USA). Rat β-arrestin 1 was amplified using specific primers and then purified. Purified product was digested and ligated into pEGFP-N1 vector between the HindIII and PstI sites to generate rat β-arrestin 1-GFP. Human β-arrestin 2 was amplified from Jurkat cDNA template. The reverse amplification primer contained a sequence encoding the flag epitope. Amplified product was purified, digested and ligated into the pEGFP-N1 vector or pcDNA3.1/Zeo (+) vector at HindIII and EcoRI sites to generate human β-arrestin 2-flag-GFP or β-arrestin 2-flag. Construction of the N-terminal HA-tagged CCR6-WT described previously (37). This construct was used as a template to generate various CCR6 mutants, in which C-terminal Ser/Thr residues were replaced with Ala through the use of a QuikChange site-directed mutagenesis kit (Stratagene). The forward and reverse primers used for amplification of β-arrestin 1, β-arrestin 2, or site-directed mutagenesis of CCR6 are shown in Table S1 in Supplementary Material.

### Cell Stimulation and ERK Phosphorylation

Jurkat cells stably expressing wild-type CCR6 (WT-CCR6) or mutant CCR6 were fasted at a density of 2 × 10^6^ cells/ml for 4–6 h in fasting medium (RPMI medium containing 0.5% BSA, 2 mM l-glutamine, 0.1 mM non-essential amino acids, 1 mM sodium pyruvate, 5.5 µM β-mercaptoethanol, and 10 mM HEPES) with or without 0.1 µg/ml pertussis toxin (PTx) (List Biological Laboratories, Campbell, CA, USA) in a 37°C incubator. Cells were then harvested, resuspended at a density of 1–2 × 10^7^ cells/ml in fasting medium, aliquoted to 100 µl of cell suspension per vial, and prewarmed on a heat block. Subsequently, cells were stimulated with 100 ng/ml CCL20 (PeproTech Inc., Rocky Hill, NJ, USA) for the indicated time periods and immediately immersed in a dry ice/ethanol bath for 3–5 s to stop the reaction. Following two washes with cold PBS, cells were lysed in lysis buffer containing 50 mM Tris–Cl, pH 7.4, 150 mM NaCl, 1% Triton, 1 mM EDTA, 1 mM EGTA, 10% glycerol, and protease inhibitor cocktail (Roche, Basel, Switzerland) and phosphatase inhibitor cocktail II (Sigma-Aldrich, St. Louis, MO, USA). Cell lysates were subjected to 9% SDS-PAGE followed by immunoblot analysis using rabbit anti-phospho ERK1/2 and mouse anti-ERK1/2, rabbit anti-phospho MEK, rabbit anti-phospho c-Raf, or rabbit anti-β-arrestin 1/2 (Cell Signaling Technology, Danvers, MA, USA). To analyze receptor desensitization, cells that were stimulated with a lower concentration of CCL20 for 3 min (WT-CCR6 and 4A-CCR6) or 10 min (3A-CCR6 and 34A-CCR6) were treated with a secondary stimulation at higher CCL20 concentration for the indicated time periods. After the stimulation was stopped, cells were lysed and subjected to 9% SDS-PAGE and immunoblot analysis.

### Receptor Internalization and Recycling

Jurkat cells stably expressing WT-CCR6 or mutant CCR6 were suspended at a density of 3 × 10^6^ cells/ml in fasting medium and stimulated with 100 ng/ml CCL20 at 37°C. Samples of the cell suspension (100 µl) were harvested at the indicated times and incubated in a dry ice/ethanol bath for 3–5 s to stop the reaction. After one wash with ice-cold FACS buffer (1% FBS, 10 mM HEPES, and 0.1% NaN_3_ in HBSS), cells were stained with PE-conjugated mouse anti-CCR6 or with an isotype control (BD Biosciences, San Jose, CA, USA). Stained cells were analyzed using a flow cytometer FACS Canto (Becton Dickinson). Internalization of CCR6 was inversely calculated as the percentage of CCR6 remaining on the cell surface after stimulation. To analyze receptor recycling, WT-CCR6 or mutant CCR6-expressing Jurkat cells were pretreated with DMSO or 10 µg/ml cycloheximide (Calbiochem, Billerica, MA, USA) in fasting medium for 15 min at 37°C followed by stimulation with 100 ng/ml CCL20 for 15 min. After washing three times, cells were further incubated in fasting medium containing DMSO or 10 µg/ml cycloheximide for the indicated time periods and then harvested for surface CCR6 staining. Data represent the mean ± SEM from at least three independent experiments.

### Chemotaxis

Chemotaxis was assayed using 6.5-mm Transwell tissue culture inserts with 5-µm pores (Corning Inc., Corning, NY, USA). Jurkat cells stably expressing WT-CCR6 or mutant CCR6 were suspended at a density of 1 × 10^7^ cells/ml in RPMI 1640 containing 10 mM HEPES and 0.5% BSA, and pre-warmed for 30 min at 37°C. One hundred microliters of cell suspension were added to the insert, which was contained in a well with 600 µl medium with or without 50 ng/ml CCL20. Cells were then incubated for 60 or 90 min at 37°C in a CO_2_ incubator. After the incubation, cells that migrated to the bottom wells were collected, pelleted and counted.

### F-Actin Polymerization Analysis

Jurkat cells stably expressing WT-CCR6 or mutant CCR6 were suspended in fasting medium at a density of 1 × 10^7^ cells/ml and aliquoted at 100 µl of cell suspension per reaction. Cells were stimulated with 100 ng/ml CCL20 for the indicated time periods and immediately fixed and stained by the addition of 1 ml of phalloidin staining buffer [1% BSA, 0.2% Triton, 4% paraformaldehyde, and 1 U of Alexa 488 tagged phalloidin (Invitrogen)] on ice for 20 min. After three washes with 0.1% Triton X-100/PBS, cells were analyzed using a flow cytometer FACS Canto. To determine cell polarization and F-actin distribution, Jurkat cells stably expressing WT-CCR6 or mutant CCR6 were seeded on 10 µg/ml fibronectin (Chemicon) coated coverslips by gravity in a 37°C incubator for 45 min. Cells were then stimulated with or without 100 ng/ml CCL20 for 10 min, fixed by 4% paraformaldehyde at room temperature for 10 min, permeabilized with 0.1% Triton/PBS for 3 min, washed three times with PBS, incubated in blocking buffer (1% BSA/PBS) for 10 min, and stained with 2.5 U/ml Alexa 488-conjugated phalloidin at room temperature for 15 min. After three washes with PBS, cells were counterstained with 0.5 µg/ml DAPI (Invitrogen) for 5 min. Coverslips were mounted on microscope slides using fluorescence mounting medium (DAKO Cytomation, Glostrup, Denmark) and viewed on an LSM700 confocal system (Carl Zeiss, Oberkochen, Germany). Two-dimensional projection images were created from z-stacks acquired using ZEN 2008 software (Carl Zeiss).

### Immunofluorescence Staining and Confocal Microscopy

U2OS cells stably expressing β-arrestin 2-GFP (6 × 10^4^) were grown on 0.1 mg/ml poly-l-lysine-coated coverslips (Sigma-Aldrich) in a 24-well plate. Cells were transfected with WT-CCR6 or various mutant CCR6 constructs for 24 h, after which they were surface stained with mouse anti-CCR6 antibody (BD Biosciences) for 20 min at room temperature, washed and then stimulated with 100 ng/ml CCL20 for indicated times at 37°C. Subsequently, cells were fixed with 4% paraformaldehyde for 10 min at room temperature, permeabilized with 0.1% Triton X-100/PBS for 5 min, washed three times with PBS, blocked with 3% BSA/PBS for 30 min, and stained with goat anti-mouse IgG conjugated with Alexa 594 (Invitrogen) for 1 h. After washing three times with PBS, cells were counterstained with 0.5 µg/ml DAPI, mounted and viewed on an LSM700 confocal system. For detection of β-arrestin 1 and CCR6, U2OS cells that had been co-transfected with the WT-CCR6 or various mutant CCR6 and β-arrestin 1-GFP constructs underwent similar experimental procedures as described earlier. For subcellular CCR6 detection, U2OS transfected with WT-CCR6 or various mutant CCR6 constructs for 24 h underwent surface CCR6 staining, CCL20 stimulation, fixation, permeabilization, and blocking as described earlier. Following CCR6 labeling, cells were stained with rabbit anti-EEA1 or rabbit anti-lamp1 (Cell Signaling Technology) for 1.5 h at room temperature, washed three times with PBS, and further incubated with goat anti-rabbit IgG F(ab′)2 conjugated with Alexa 488 and goat anti-mouse IgG conjugated with Alexa 594 (Invitrogen) for 1 h at room temperature. Cells were counterstained with DAPI and observed on an LSM700 confocal system.

### Immunoprecipitation of HA-CCR6

HEK293T cells (2 × 10^6^) were seeded in 6 cm-dish and co-transfected with HA-tagged WT-CCR6 or various mutant CCR6 and β-arrestin 1-flag, β-arrestin 2-flag, or β-arrestin 2-flag-GFP for 24 h. Cells were trypsinized, resuspended at a density of ~1 × 10^7^ cells/ml in fasting medium, stimulated with 100 ng/ml CCL20 for the indicated time, incubated in a dry ice/ethanol bath to stop reaction and lysed with lysis buffer for 30–60 min on ice. After centrifugation at 12,000 × *g* for 10 min, supernatants (~1 mg) were pre-cleared with protein A agarose beads (Millipore, Billerica, MA, USA) for 1 h at 4°C. Pre-cleared lysates were incubated with anti-HA affinity gel (Sigma-Aldrich) for 3 h at 4°C and the immunoprecipitates were washed, denatured with Laemmli buffer for 30 min at 50–55°C and subjected to 9% SDS-PAGE, followed by western blotting with rabbit anti-flag M2 (Sigma-Aldrich) or Rat anti-HA 3F10 (Roche).

### Phospho-Labeling

HEK293T cells (5 × 10^6^) in 10 cm-dish were transfected with HA-tagged WT-CCR6 or AA-CCR6 constructs for 24 h. Cells were fasted in phosphate-free DMEM medium containing 0.5% BSA and 10 mM HEPES for 3 h at 37°C, after which they were trypsinized and resuspended in phosphate-free DMEM at a density of 1 × 10^7^ cells/ml. Cells were metabolically labeled with 60 μCi/ml of P^32^ for 1.5 h at 37°C followed by stimulation with 100 ng/ml CCL20 for 0 or 5 min. Cells were washed, lysed with RIPA buffer, and subjected to immunoprecipitation of HA-tagged CCR6 using anti-HA affinity gel as described earlier. The immunoprecipitates were run on 9% SDS-PAGE and P^32^ signals were detected and quantified by Typhoon 9410 image system (Amersham BioScience, Piscataway, NJ, USA).

### Statistical Analysis

Unpaired Student’s *t*-tests were applied to evaluate the differences between experimental groups. *p* ≤ 0.05 was considered statistically significant.

## Results

### Ser/Thr Residues within the C-Terminal Domain of CCR6 Are Phosphorylated upon CCL20 Stimulation

**Figure 1:**
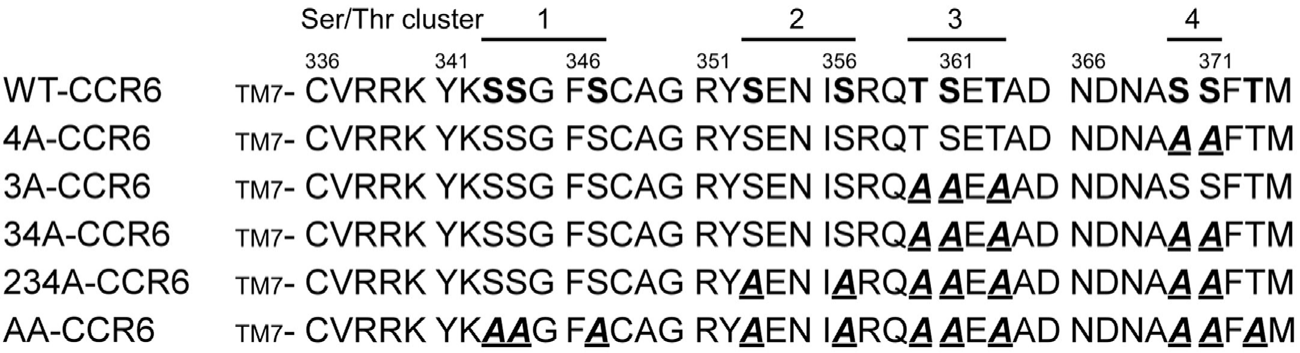
C-terminal protein sequences for wild-type (WT) and various CCR6 mutants. C-terminal Ser/Thr residues of WT CCR6 and various CCR6 C-terminal mutants, wherein C-terminal Ser/Thr residues were replaced with Ala as indicated: 3A-CCR6 (T360A, S361A, and T363A), 4A-CCR6 (S370A and S371A), 34A-CCR6 (T360A, S361A, T363A, S370A, and S371A), 234A-CCR6 (S353A, S357A, T360A, S361A, T363A, S370A, and S371A), and AA-CCR6 (all C-terminal Ser/Thr residues were replaced with Ala).

Ligand binding to GPCRs induces the phosphorylation of Ser/Thr residues in the C-terminal tails of the receptors (38). Recent studies have shown that specific phosphorylation patterns in these C-terminal domains lead to distinct biological effects (25, 26, 28, 39). To determine the C-terminal Ser/Thr residues that are phosphorylated in CCL20-bound CCR6, we metabolically labeled HA-tagged CCR6 transfectants with [^32^P]-orthophosphate and stimulated cells with CCL20 followed by immunoprecipitation of CCR6 with anti-HA. The phosphorylation of WT-CCR6 was readily detected upon CCL20 stimulation (Figure S1 in Supplementary Material). However, the phosphorylation of mutant CCR6 (AA-CCR6), in which all Ser/Thr residues in the C-terminal tail were mutated into Ala, was completely absent. This result indicates that the CCL20-induced phosphorylation of CCR6 is limited to Ser/Thr residues in the C-terminal tail and does not extend to residues within intracellular loops. To study the effect of C-terminal Ser/Thr residue clusters on CCR6 biological function, we generated a series of CCR6 C-terminal Ser/Thr mutants, shown in Figure 1. We grouped the 11 Ser/Thr residues at the C-terminus of CCR6 into four clusters, designated 1–4 (ordered from the N- to C-terminus). Various CCR6 mutants were constructed with the grouped Ser/Thr residues systematically replaced by Ala. In the 3A-CCR6 construct, Thr360, Ser361, and Thr363 (cluster 3) were mutated to Ala. For the 4A-CCR6 construct, Ser370 and Ser371 (cluster 4) were mutated to Ala. For the 34A-CCR6 construct, Ser/Thr residues in both cluster 3 and cluster 4 were mutated to Ala. For the 234A-CCR6 construct, Ser-353 and Ser-357 (cluster 2) plus Ser/Thr residues in cluster 3 and cluster 4 were mutated to Ala. For the AA-CCR6, all C-terminal Ser/Thr residues were mutated to Ala. To make a more valid comparison of biological effects among WT-CCR6 and CCR6 mutants, we established stable clones for each CCR6 mutant in Jurkat cells by choosing clones that expressed comparable levels of surface receptor (Figure S2 in Supplementary Material).

### Ser/Thr Residue Clusters in the C-Terminal Domain of CCR6 Differentially Regulate CCL20-Induced CCR6 Internalization and Recycling

**Figure 2:**
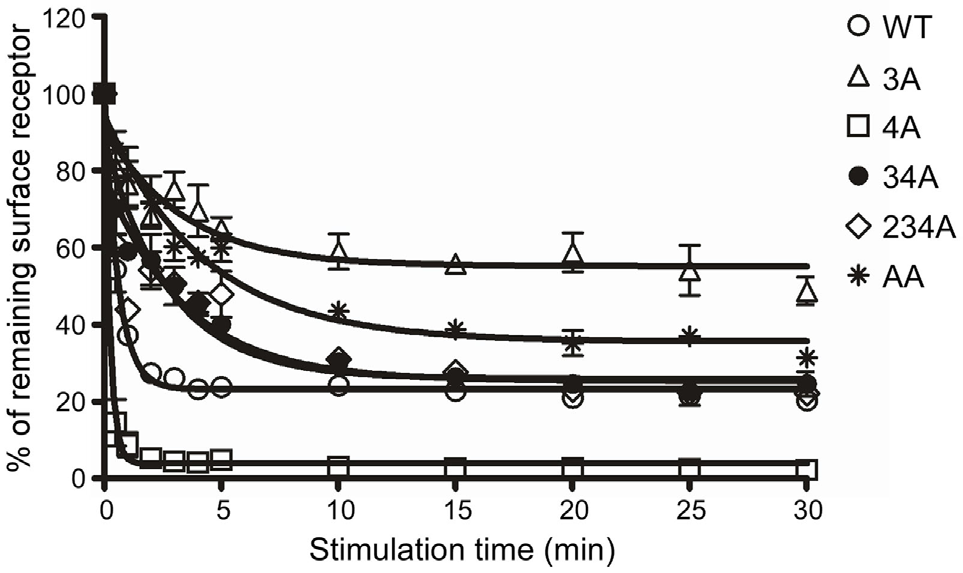
C-terminal Ser/Thr clusters in CCR6 differentially regulate CCL20-induced CCR6 internalization. Jurkat cells stably expressing wild-type CCR6 or various CCR6 mutants were stimulated with 100 ng/ml CCL20 for the times indicated. Cells were washed with ice-cold FACS buffer and stained with PE-conjugated mouse anti-CCR6 or with an isotype control. Surface receptor levels were measured using flow cytometry. Internalization of CCR6 is reflected by the percentage of CCR6 remaining on surface after stimulation.

Receptor phosphorylation on the C-terminal tail is known to be a prerequisite for the internalization of GPCRs (40). To identify C-terminal Ser/Thr residues that are critical for CCR6 internalization, we treated WT-CCR6 and various CCR6 mutants with CCL20 for up to 30 min. The kinetics of receptor internalization were assessed by determining the loss of surface receptor using flow cytometry. Surface 4A-CCR6 was internalized most rapidly and efficiently among CCR6 mutants (Figure 2). Approximately 91.14 ± 3.14% of the 4A-CCR6 surface receptor was internalized as early as 1 min after CCL20 stimulation, an effect which was sustained at least for 30 min (Figure 2). Even when compared with WT-CCR6, 4A-CCR6 showed much more efficient internalization. On the other hand, 3A-CCR6, 34A-CCR6, 234A-CCR6, and AA-CCR6 mutants exhibited slower internalization kinetics when compared with WT-CCR6 or 4A-CCR6 (Figure 2). The relative efficiency of receptor internalization for the constructs was: 4A-CCR6 > WT-CCR6 > 234A-CCR6 ≈ 34A-CCR6 > AA-CCR6 > 3A-CCR6 (Figure 2). Notably, the kinetics of receptor internalization reached a stationary phases after stimulation with CCL20 for 1 min in 4A-CCR6, 4 min in WT-CCR6, and 15–20 min in 3A-CCR6, 34A-CCR6, 234A-CCR6, and AA-CCR6-expressing cells. The stationary phases may result from the steady-state balance of several variables, including the rates of internalization, recycling, degradation or synthesis of receptors. Taken together, the data showed that phosphorylation of cluster 3 (Thr360, Ser361, and Thr363) is critical for CCL20-induced CCR6 internalization, while the phosphorylation of cluster 4 (Ser370 and Ser371) hinders CCL20-induced CCR6 internalization.

**Figure 3:**
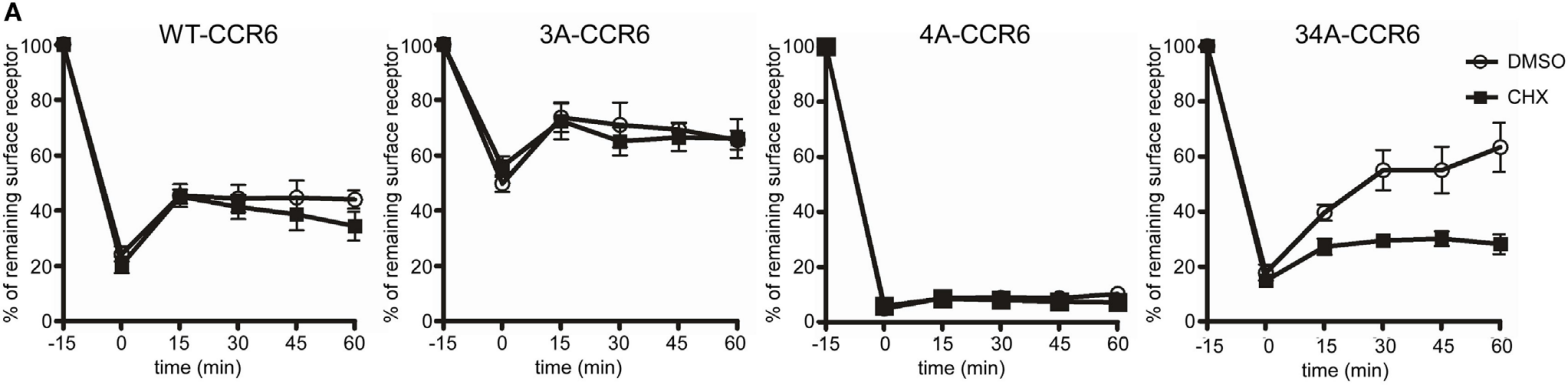

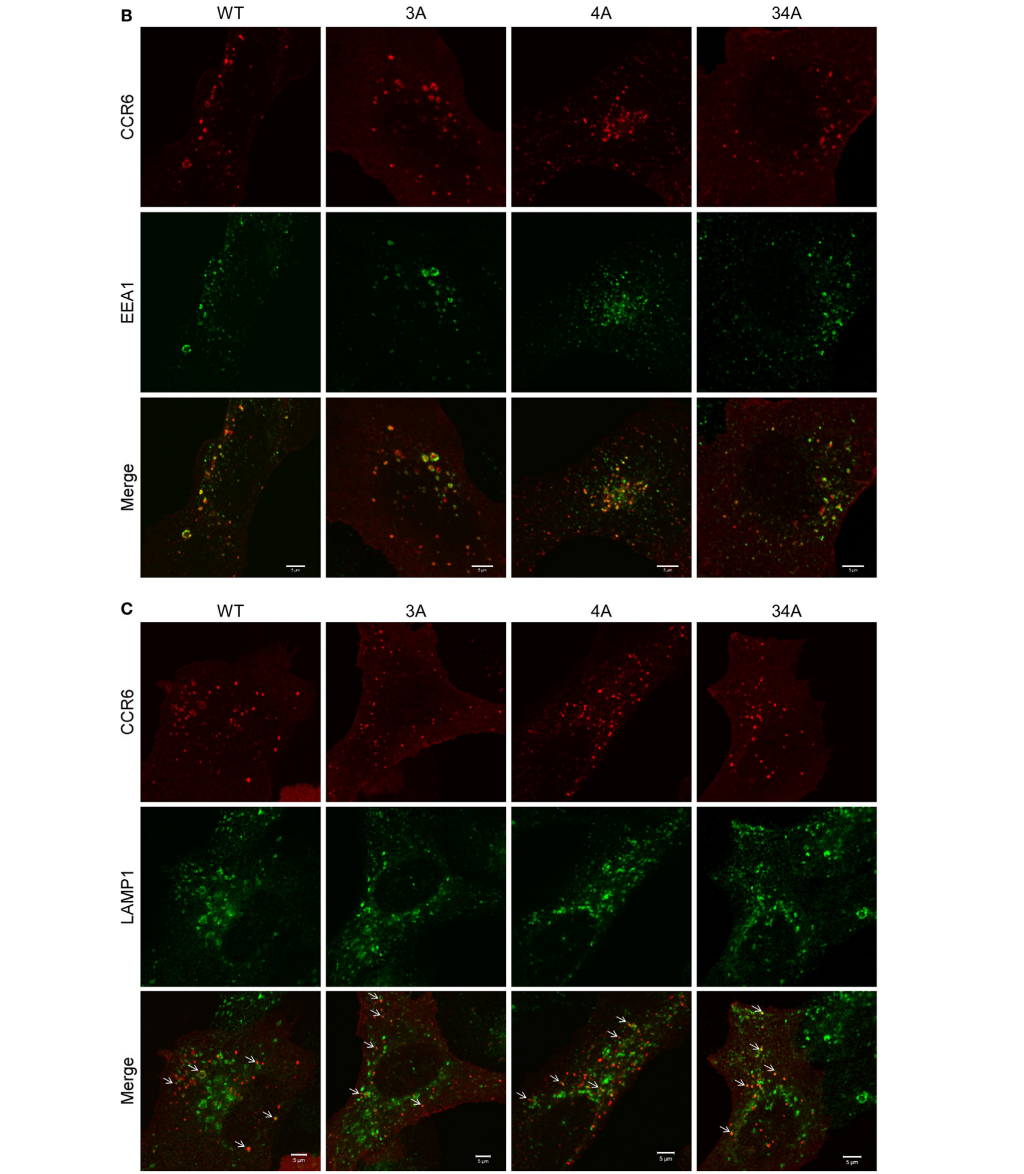
The destination of internalized wild-type CCR6 (WT-CCR6) and CCR6 mutant proteins. **(A)** Jurkat cells stably expressing WT-CCR6 or various CCR6 mutants were pretreated with DMSO or 10 µg/ml cycloheximide for 15 min at 37°C followed by stimulation with 100 ng/ml CCL20 for 15 min. After washing, cells were further incubated in medium containing DMSO or 10 µg/ml cycloheximide for the indicated times. Cells were then harvested and subjected to surface CCR6 staining. Surface levels of CCR6 were quantified by flow cytometry. Data represent the mean ± SEM from four independent experiments. **(B,C)** U2OS cells were transfected with WT-CCR6 or various CCR6 mutant constructs for 24 h. Cells were then stimulated with CCL20 for 5 or 20 min, fixed, permeabilized, and stained for human CCR6 (red) along with rabbit anti-EEA1 [green, **(B)**] or rabbit anti-lamp1 [green, **(C)**]. Cells were observed using LSM700 confocal system. Scale bar = 5 µm.

Following internalization, receptors are either recycled to the cell surface or targeted to lysosomes for degradation. To investigate the effects of C-terminal Ser/Thr residues on CCR6 intracellular trafficking, we stimulated WT-CCR6 or various CCR6 mutant-expressing transfectants with CCL20 and compared the surface receptor levels in the presence or absence of the protein synthesis inhibitor, cycloheximide (CHX). Transfectants expressing WT-CCR6 or various CCR6 mutants were subjected to CCL20 stimulation for 15 min, followed by the removal of CCL20 and further incubation for the indicated times. After CCL20 was removed, the patterns of receptor trafficking were not affected by CHX in cells expressing WT-CCR6, 3A-CCR6, or 4A-CCR6 (Figure 3A), suggesting that the reappearance of these receptors on the cell surface does not require new protein synthesis. However, only a minor portion of receptors were recycled, and recycling occurred to different extents for different constructs (WT-CCR6: 25%, 3A-CCR6: 16.5%, and 4A-CCR6: 2.6%). Also, after an initial burst of recycling, the surface levels of protein were sustained throughout the rest of the experiments (Figure 3A). In contrast to these three constructs, the surface levels of 34A-CCR6 were gradually increased in vehicle-treated cells, while CHX-treated cells showed a surface expression profile that was similar to WT-CCR6, 3A-CCR6, and 4A-CCR6 (12.3% receptors were recycled). This result suggests that the reappearance of 34A-CCR6 on the cell surface requires *de novo* protein synthesis. From these data, we conclude that WT-CCR6 and CCR6 mutants are partially recycled and that Ser/Thr residues in cluster 3 and cluster 4 may work in concert to prevent *de novo* protein synthesis of CCR6. In addition, immunofluorescence staining revealed that almost all of the observable internalized WT-CCR6, 3A-CCR6, 4A-CCR6, and 34A-CCR6 proteins were targeted to EEA1^+^ early endosomes (Figure 3B), with some signal colocalizing with LAMP1^+^ lysosomes (indicated by arrows, Figure 3C). Taken together, our results indicate that CCR6 receptors are partially recycled to the cell surface, and some internalized CCR6 is further targeted to lysosomal degradation. Moreover, the different clusters of Ser/Thr residues in the C-terminal domain of CCR6 regulate the extent of receptor recycling to some degree.

### Ser/Thr Residue Clusters Differentially Regulate CCL20-Induced Chemotaxis

**Figure 4:**
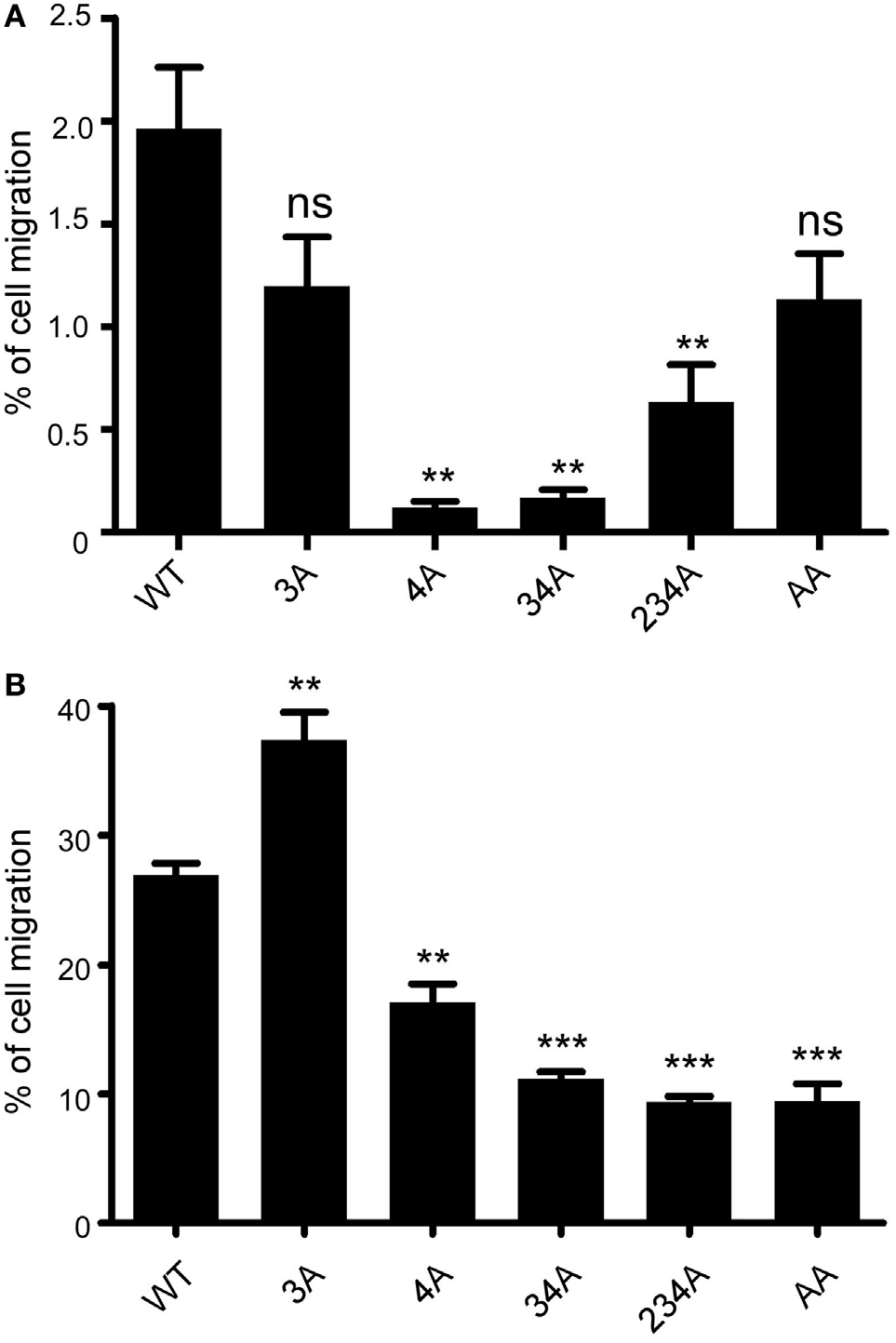
CCR6 C-terminal Ser/Thr clusters differentially regulate CCL-20-induced chemotaxis. **(A)** Jurkat cells stably expressing wild-type CCR6 (WT-CCR6) or various CCR6 mutants were analyzed by transwell migration assays. The data are expressed as percentage of input cells that migrated to the lower chamber in the absence of CCL20. **(B)** WT-CCR6 or various CCR6 transfectants were analyzed by transwell migration assays. The data are expressed as percentage of input cells that migrated to the lower chamber containing 50 ng/ml CCL20. Data represent the mean ± SEM from four independent experiments (**p < 0.01 and ***p < 0.001 vs. WT-CCR6).

A major biological function of chemokine receptors is to stimulate leukocyte migration into infected and/or inflamed tissues (41). Many studies have demonstrated that chemokine receptor signaling activates small GTPase and actin polymerization, which collectively regulate leukocyte migration. We first examined whether different CCR6 mutants showed different degree of random migration. Performing a chemotaxis assay in the absence of CCL20, we found that the random migration of different CCR6 mutants varied (Figure 4A). The random migration of various transfectants was less then 2% of input cells; however, 4A-CCR6, 34A-CCR6, and 234A-CCR6 transfectants showed significantly reduced random migration compared with WT-CCR6 transfectants, while 3A-CCR6 transfectants showed similar random migration as WT-CCR6 transfectants (Figure 4A). We then performed a chemotaxis assay in the presence of CCL20 and found that 3A-CCR6 transfectants displayed an enhanced chemotactic activity, while 4A-CCR6 transfectants showed reduced chemotactic activity compared with WT-CCR6 transfectants (Figure 4B). These results suggest that Ser/Thr residues in cluster 3 and cluster 4 of CCR6 oppositely regulate cell migration. We observed that all transfectants with cluster 4 mutations (4A-CCR6, 34A-CCR6, 234A-CCR6, and AA-CCR6) showed reduced chemotaxis activity as compared with WT-CCR6 transfectants (Figure 4B), regardless of whether cluster 3 was mutated. Since cluster 4 mutations were able to reverse the increased cell migration from cluster 3 mutations, we infer that Ser/Thr residues in cluster 4 may predominately regulate cell migration. Because 4A-CCR6, 34A-CCR6, and 234A-CCR6 transfectants showed reduced random migration (Figure 4A) as well as reduced directional migration (Figure 4B), the reduced random migration might also contribute, in part, to the reduced chemotactic activity.

### Ser/Thr Residue Clusters Differentially Regulate CCL20-Induced Actin Polymerization

**Figure 5:**
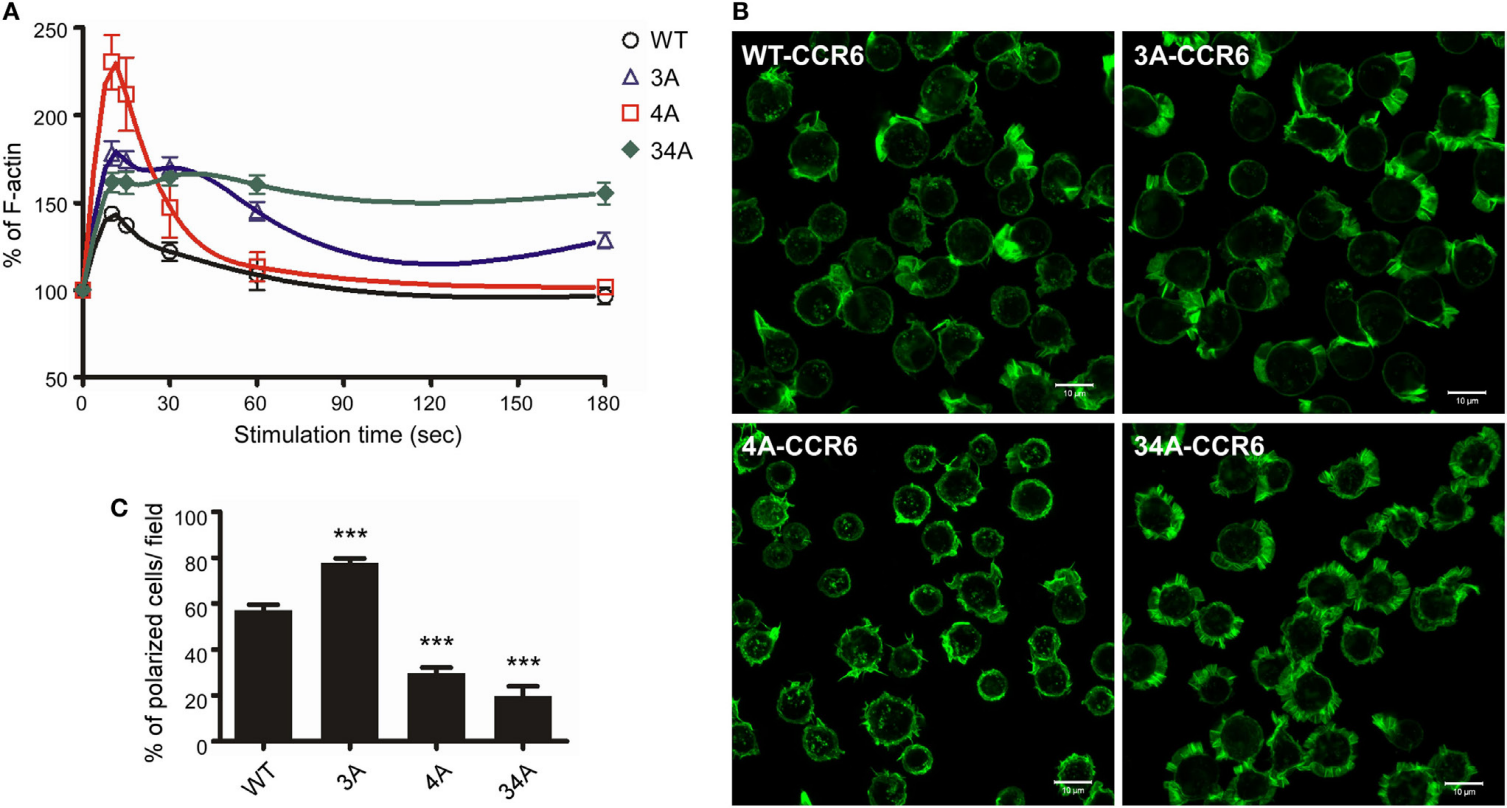
CCR6 C-terminal Ser/Thr clusters affect CCL20-induced actin polymerization. **(A)** Jurkat cells stably expressing wild-type CCR6 (WT-CCR6) or various CCR6 mutants were stimulated with 100 ng/ml CCL20 for the indicated times, fixed by 4% formaldehyde, stained with phalloidin conjugated with Alexa 488, and subjected to flow cytometry analysis. **(B)** Jurkat cells stably expressing WT-CCR6 or various CCR6 mutants were seeded on 10 µg/ml fibronectin-coated coverslips and stimulated with 100 ng/ml CCL20 for 10 min, fixed by 4% formaldehyde, and stained with Alexa 488-conjugated phalloidin. Images were obtained on an LSM700 confocal microscope. Scale bar = 10 µm. **(C)** Percentage of polarized cells per field was calculated (*n* = 6–24). ***p < 0.001 compared with WT-CCR6.

For directional migration to occur, actin polymerization must be regulated to create cell protrusions at the leading edge (42). Given our observation that C-terminal Ser/Thr residues regulated chemotaxis (Figure 4), we hypothesized that the phosphorylation of the CCR6 C-terminal tail may be involved in regulating actin polymerization. We measured the global F-actin levels in transfectants expressing WT-CCR6 and various CCR6 mutants using phalloidin staining followed by FACS analysis. Stimulating WT-CCR6 or various mutant CCR6 receptors with CCL20-induced rapid actin polymerization, peaking at 15 s; however, the kinetics and magnitude of F-actin accumulation varied among the constructs (Figure 5A). Compared with WT-CCR6 transfectants, the level of F-actin in 3A-CCR6 transfectants was more rapidly increased and still peaked at 15 s, but the peak level was sustained until 30 s and then very gradually declined (Figure 5A). The sustained actin polymerization in 3A-CCR6 transfectants may be associated with the increased chemotactic activity shown in Figure 4. The levels of F-actin in 4A-CCR6 transfectants were transiently increased, with a strong peak at 15 s and a rapid decline. The maximal magnitude was approximately three-fold greater than that of WT-CCR6 transfectants. Although 4A-CCR6 transfectants displayed a higher magnitude of actin polymerization than WT-CCR6 transfectants, the same 4A-CCR6 transfectants had reduced chemotactic activity. Interestingly, the levels of F-actin in 34A-CCR6 were rapidly increased and remained elevated throughout the experiment (Figure 5A), suggesting that 34A-CCR6 transfectants failed to undergo actin depolymerization. Disturbing the dynamics of actin polymerization and depolymerization in 34A-CCR6 transfectants may contribute to the observed impairment in chemotactic activity. Taken together, the altered random cell migration as well as directional cell migration (Figure 4) indeed associate with the altered actin dynamics of 3A-CCR6, 4A-CCR6, and 34A-CCR6 transfectants (Figure 5).

To further investigate whether the actin polymerization in WT-CCR6 and various mutant CCR6 transfectants affected cell polarization upon CCL20 stimulation, we analyzed intracellular F-actin distribution with confocal microscopy after phalloidin staining (Figure 5B). WT-CCR6 and various mutant CCR6 transfectants were seeded on fibronectin-coated coverslips and stimulated with CCL20 for 10 min. In the absence of CCL20 stimulation, the basal levels of F-actin were equally distributed in the periphery (data not shown). Upon CCL20 stimulation, WT-CCR6 and 3A-CCR6 transfectants induced the formation of lamellipodia, in which F-actin was concentrated at the leading edge (Figure 5B). The formation of this type of leading edge is expected to effectively promote directional migration. Interestingly, we found that 4A-CCR6 transfectants displayed compromised lamellipodium structure with thinner lamella, which might explain the relative inability of these tranfectants to migrate. Furthermore, 34A-CCR6 transfectants displayed strong actin polymerization that was evenly distributed around the cell periphery and thus failed to form a leading edge to promote directional migration (Figure 5B). The percentage of polarized cells with lamellipodia formation were quantified from each group, producing data that mirrored the chemotaxis profiles for the transfectants (Figure 5C). Together, these results suggest that Ser/Thr residues in the C-terminal domain of CCR6 can impact F-actin formation/accumulation to control directional cell migration. Furthermore, the Ser/Thr residues in cluster 3 and cluster 4 appear to work in concert to dynamically and spatiotemporally regulate actin polymerization and depolymerization to promote cell migration.

### Ser/Thr Residue Clusters Differentially Regulate CCL20-Induced ERK1/2 Phosphorylation

**Figure 6:**
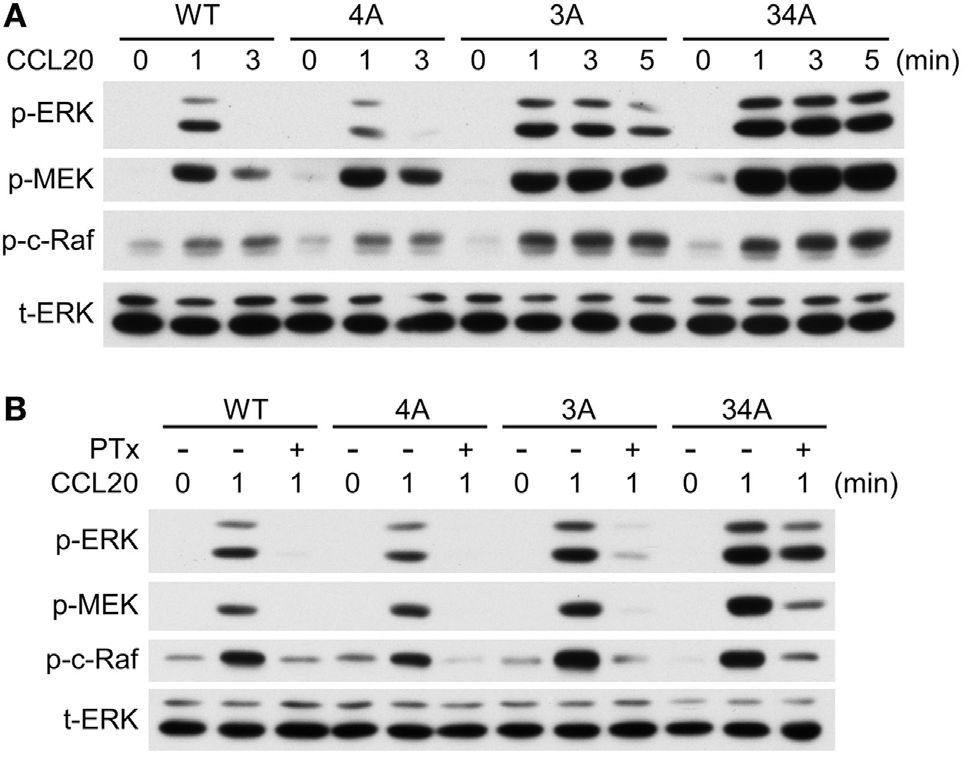
CCR6 C-terminal Ser/Thr clusters differentially regulate CCL20-induced ERK1/2 phosphorylation. **(A)** Jurkat cells stably expressing wild-type CCR6 (WT-CCR6) or various CCR6 mutants were fasted and then stimulated with 100 ng/ml CCL20 for 0, 1, 3, or 5 min. Cell lysates were analyzed by immunoblotting using anti-phospho-ERK1/2 (ERK-p), anti-total ERK, anti-phospho-MEK (MEK-p), and anti-phospho-c-Raf (c-Raf-p). **(B)** Jurkat cells stably expressing WT-CCR6 or various CCR6 mutants were pretreated with 100 ng/ml pertussis toxin for 4 h followed by stimulation with 100 ng/ml CCL20. Lysates were subjected to immunoblotting analysis.

To determine whether the phosphorylation of CCR6 C-terminal Ser/Thr residues affects the activation of known signaling effectors, we examined ERK1/2 activation in WT-CCR6 and various mutant CCR6 transfectants after CCL20 stimulation. Western blot analysis showed that CCL20-mediated ERK1/2 phosphorylation peaked at 1 min in WT-CCR6 and mutant CCR6 transfectants. The intensity and persistence of ERK1/2 activation varied among the transfectants (Figure 6A; Figure S3 in Supplementary Material). ERK1/2 activation was transiently induced in WT-CCR6 and 4A-CCR6 transfectants, with no signal detected as early as 3 min after stimulation. However, in 3A-CCR6 transfectants, ERK1/2 activation gradually declined after peaking at 1 min. Strikingly, ERK1/2 phosphorylation was sustained at peak levels for at least 5 min in 34A-CCR6 transfectants (Figure 6A). We then examined the activation of signaling molecules upstream of ERK activation. The CCL20-induced activation of MEK and c-Raf was largely consistent with CCL20-induced ERK1/2 activation (Figure 6A).

Chemokine receptor signaling is mediated predominantly through coupling to the Gαi family of heterotrimeric G proteins (41, 43). Therefore, we used PTx, an inhibitor of Gαi coupling to GPCRs, to examine whether CCR6 C-terminal Ser/Thr mutants require Gαi to activate ERK1/2. As expected, PTx pretreatment completely abolished CCL20-mediated ERK1/2 activation in WT-CCR6 transfectants (Figure 6B), confirming that this action is Gαi-dependent. Similarly, Gαi-dependent ERK1/2 activation was also observed in 4A-CCR6 transfectants. Interestingly, PTx failed to completely inhibit CCL20-induced ERK1/2 activation in 3A-CCR6 and 34A-CCR6 transfectants, and this loss of Gαi dependence was particularly apparent in 34A-CCR6 transfectants (Figure 6B). Thus, an inability to phosphorylate Ser/Thr residues in cluster 3, or in cluster 3 and cluster 4, may introduce some independence from the Gαi signaling pathway, which may potentially result in the sustained signaling observed in Figure 6A. Overall, these results suggest that phosphorylation of Ser/Thr residues in cluster 3 (Thr360, Ser361, and Thr363) may regulate the termination of CCR6 signaling, and that cluster 4 (Ser370 and Ser371) may enhance the termination signal. Furthermore, since the onset of ERK1/2 activation was not measurably affected in the mutants, the phosphorylation of CCR6 C-terminal Ser/Thr clusters may only act to terminate the signaling and not affect the initiation of CCL20-mediated ERK1/2 activation.

### CCR6 C-Terminal Ser/Thr Clusters Differentially Affect Receptor Desensitization

**Figure 7:**
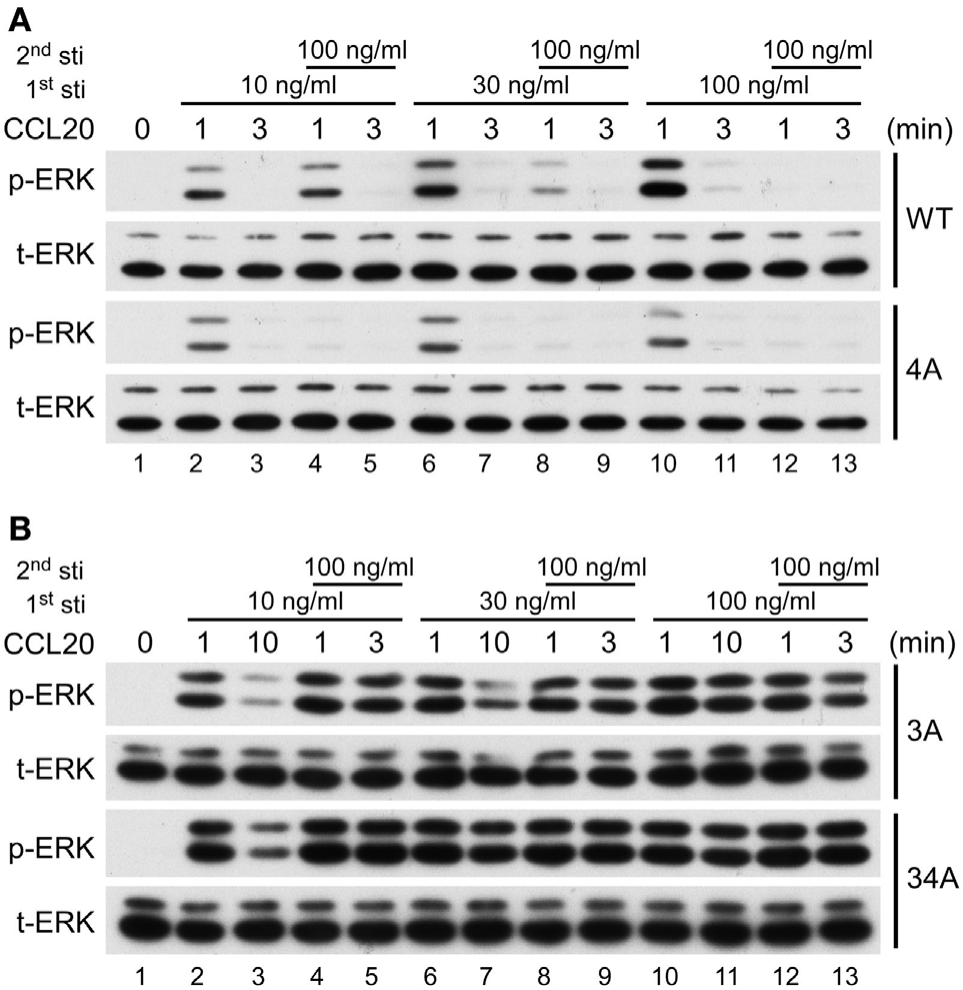
CCR6 C-terminal Ser/Thr clusters differentially regulate CCL 20-induced desensitization. Jurkat cells stably expressing wild-type CCR6, 4A-CCR6 **(A)**, 3A-CCR6 or 34A-CCR6 **(B)** were stimulated with 10, 30, or 100 ng/ml CCL20 for 0, 1, or 3 min. Three minutes **(A)** or 10 min **(B)** after the first stimulation, the cells were subjected to a second stimulation with 100 ng/ml CCL20 for 1 or 3 min. After the second stimulation, cell lysates were collected and used for immunoblotting with anti-phospho ERK1/2 (ERK-p) and anti-total ERK (tERK) antibodies.

It is well known that upon ligand stimulation, the phosphorylation of Ser/Thr residues in the C-terminal domain of GPCRs leads to receptor desensitization (22–24). We investigated which clusters of CCR6 C-terminal Ser/Thr residues are responsible for CCR6 desensitization. Because CCL20-stimulated WT-CCR6 and various CCR6 mutants to induce Gαi-mediated ERK1/2 phosphorylation (Figure 6B), we examined receptor desensitization by measuring ERK1/2 activation. The kinetics of CCL20-mediated induction of ERK1/2 activation in WT-CCR6 and various mutant CCR6 transfectants were variable and the time required to turn off ERK1/2 phosphorylation also varied among transfectants (Figure 6A). Therefore, we designed desensitization experiments with these differences in mind. For WT-CCR6 and 4A-CCR6 transfectants, cells were stimulated with CCL20 for 3 min followed by a second stimulation. On the other hand, 3A-CCR6 and 34A-CCR6 transfectants were stimulated with CCL20 for a longer time (10 min) to allow the signal to diminish before the second stimulation. Transfectants were initially stimulated for with 10, 30, or 100 ng/ml CCL20, or left untreated. CCL20 (100 ng/ml) was then introduced for the second stimulation and the degree of receptor desensitization was determined by measuring ERK1/2 phosphorylation levels. Transfectants that received only the first CCL20 stimulation showed the anticipated patterns of ERK1/2 phosphorylation (Figure 7, lanes 1, 2, 6, and 10). After the second stimulation with 100 ng/ml CCL20, WT-CCR6 transfectants that were initially stimulated with 10, 30, or 100 ng/ml exhibited reductions in ERK1/2 activity of 63.4, 86.9, and 99.9%, respectively (Figure 7A, lane 4 vs. 10; lane 8 vs. 10; lane 12 vs. 10). Interestingly, 4A-CCR6 transfectants that were initially stimulated with 10, 30, or 100 ng/ml CCL20 and then received a second stimulation with 100 ng/ml CCL20 exhibited a complete loss of ERK1/2 activity, suggesting that Ser/Thr residues in cluster 4 play a role in desensitization (Figure 7A, lower blots). By contrast, 3A-CCR6 and 34A-CCR6 failed to show evidence of desensitization upon the second CCL20 stimulation (Figure 7B, lane 2 vs. 4; lane 6 vs. 8; lane 10 vs. 12), suggesting that Ser/Thr residues in cluster 3 are required for CCL20-mediated receptor desensitization. Taken together, these results suggest that phosphorylation of C-terminal cluster 4 regulates receptor desensitization, whereas phosphorylation of C-terminal cluster 3 contributes to receptor desensitization.

### CCR6 Phosphorylation Recruits β-Arrestins

**Figure 8:**
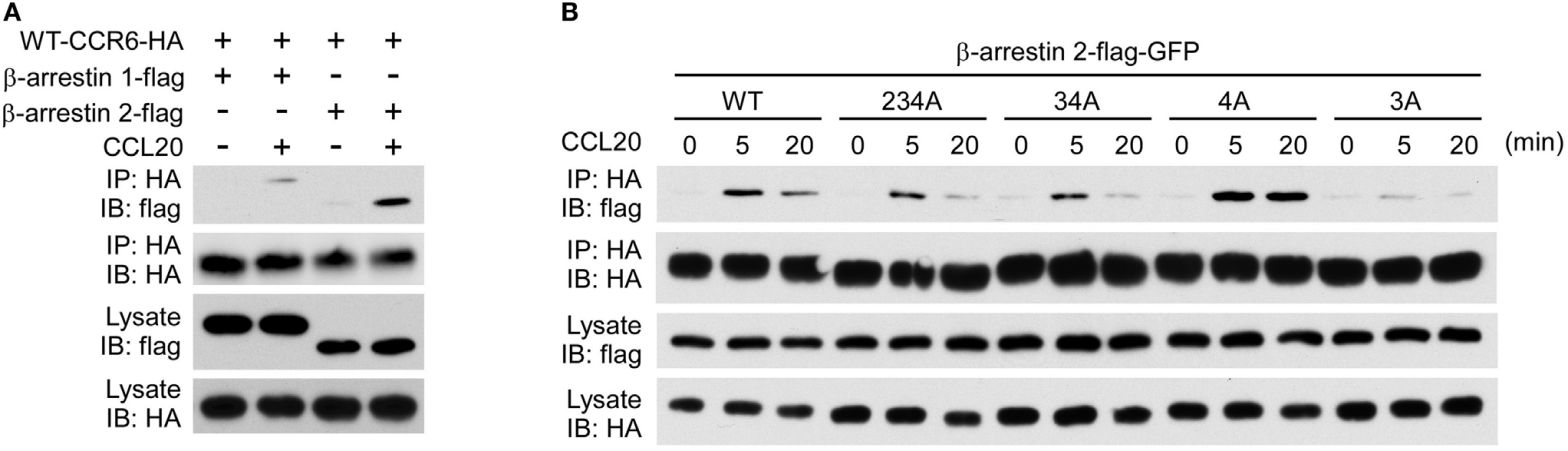

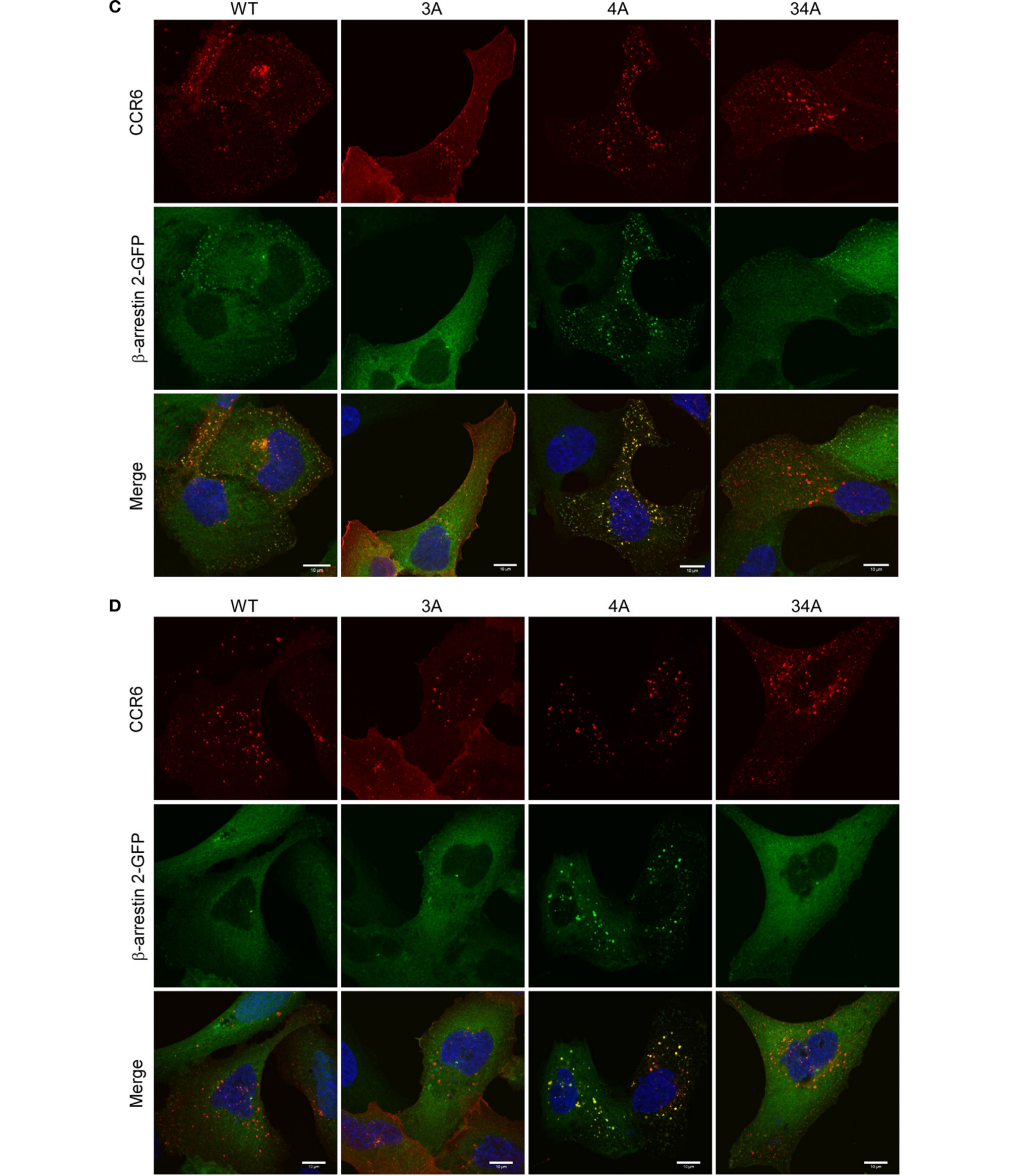
CCR6 C-terminal Ser/Thr clusters are critical for interaction with β-arrestin 2. **(A)** HEK293T cells were transfected with HA-wild-type CCR6 (WT-CCR6) along with β-arrestin 1-flag or β-arrestin 2-flag. After 24 h, the cells were then stimulated with 100 ng/ml CCL20 for 0 or 5 min. After stimulation, cells were lysed, and cell lysates were immunoprecipitated with anti-HA agarose followed by western blotting for the flag and HA tags. **(B)** HEK293T cells were transfected with pEGFP-β-arrestin 2-flag along with HA-tagged WT-CCR6 or various CCR6 mutants. After 24 h, cells were stimulated with CCL20 for 0, 5, or 20 min. After CCL20 stimulation, cells were collected and lysed followed by immunoprecipitation and immunoblotting. **(C)** U2OS cells stably expressing β-arrestin 2-GFP (green) were transfected with WT-CCR6 and various CCR6 mutants for 24 h. Cells were stained with anti-CCR6 antibody followed by stimulation with 100 ng/ml CCL20 for 5 min **(C)** or 20 min **(D)**. After stimulation with CCL20, cells were fixed and stained with anti-mouse-Alexa 594 (red) and DAPI (blue). Images were obtained by LSM700 confocal microscopy. Scale bar = 10 µm.

The recruitment of non-visual arrestins, β-arrestin 1 and β-arrestin 2, to phosphorylated GPCRs is important for receptor internalization, desensitization and signaling transduction (22, 44). We used immunoprecipitation and immunofluorescence assays to examine whether β-arrestin 1 and β-arrestin 2 were recruited to CCR6 upon CCL20 stimulation. HEK293T cells were co-transfected with plasmid encoding HA-tagged WT-CCR6 and another plasmid encoding β-arrestin 1-flag or β-arrestin 2-flag. The transfectants were stimulated with CCL20, and after harvesting, cell lysates were subjected to immunoprecipitation of CCR6 using anti-HA agarose, followed by western blotting for β-arrestin 1-flag and β-arrestin 2-flag. Western blot analysis showed that activated CCR6 recruited both β-arrestin 1-flag and β-arrestin 2-flag; however, the CCR6 interaction with β-arrestin 2-flag was stronger than the interaction with β-arrestin 1-flag (Figure 8A). We further examined which clusters of Ser/Thr residues are critical for the recruitment of β-arrestin 2. Lysates from CCL20-stimulated HEK293T transfectants co-expressing β-arrestin 2-flag-GFP and HA-tagged WT-CCR6 or various CCR6 mutants were subjected to the immunoprecipitation assay. β-Arrestin 2-flag-GFP showed stronger and more sustained association with 4A-CCR6 compared with WT-CCR6. However, the association of β-arrestin 2-flag-GFP with 3A-CCR6, 34A-CCR6, or 234A-CCR6 was weaker than its association with WT-CCR6. In particular, 3A-CCR6 showed the weakest association with β-arrestin 2-flag-GFP (Figure 8B).

We further confirmed the association of CCR6 with β-arrestin 2 by immunofluorescence analysis. U2OS cells that stably expressed β-arrestin 2-GFP were transfected with expression plasmids encoding WT-CCR6 or various mutant CCR6 receptors. The transfectants were stained with fluorescence conjugated anti-CCR6 antibody followed by CCL20 stimulation. After stimulation, cells were fixed, stained for CCR6 and observed under a confocal microscope. Upon CCL20 stimulation, β-arrestin 2-GFP formed punctate structures that were colocalized with internalized WT-CCR6 (Figure 8C). CCL20-stimulated 4A-CCR6 recruited β-arrestin 2-GFP more thoroughly and formed more stable β-arrestin 2-GFP punctate structures (Figures 8C,D) compared with CCL20-stimulated WT-CCR6. By contrast, β-arrestin 2-GFP was poorly recruited by either 3A-CCR6 or 34A-CCR6 upon CCL20 stimulation (Figure 8C). Taken together, these results suggest that C-terminal cluster 3 (Thr360, Ser361, and Thr363) is important for the recruitment of β-arrestin 2 to phosphorylated CCR6 upon CCL20 stimulation, while the phosphorylation of cluster 4 (Ser370 and Ser371) seems to prevent the recruitment of β-arrestin 2.

### β-Arrestin 1 and β-Arrestin 2 Play Distinct Roles in CCR6-Mediated Signaling

**Figure 9:**
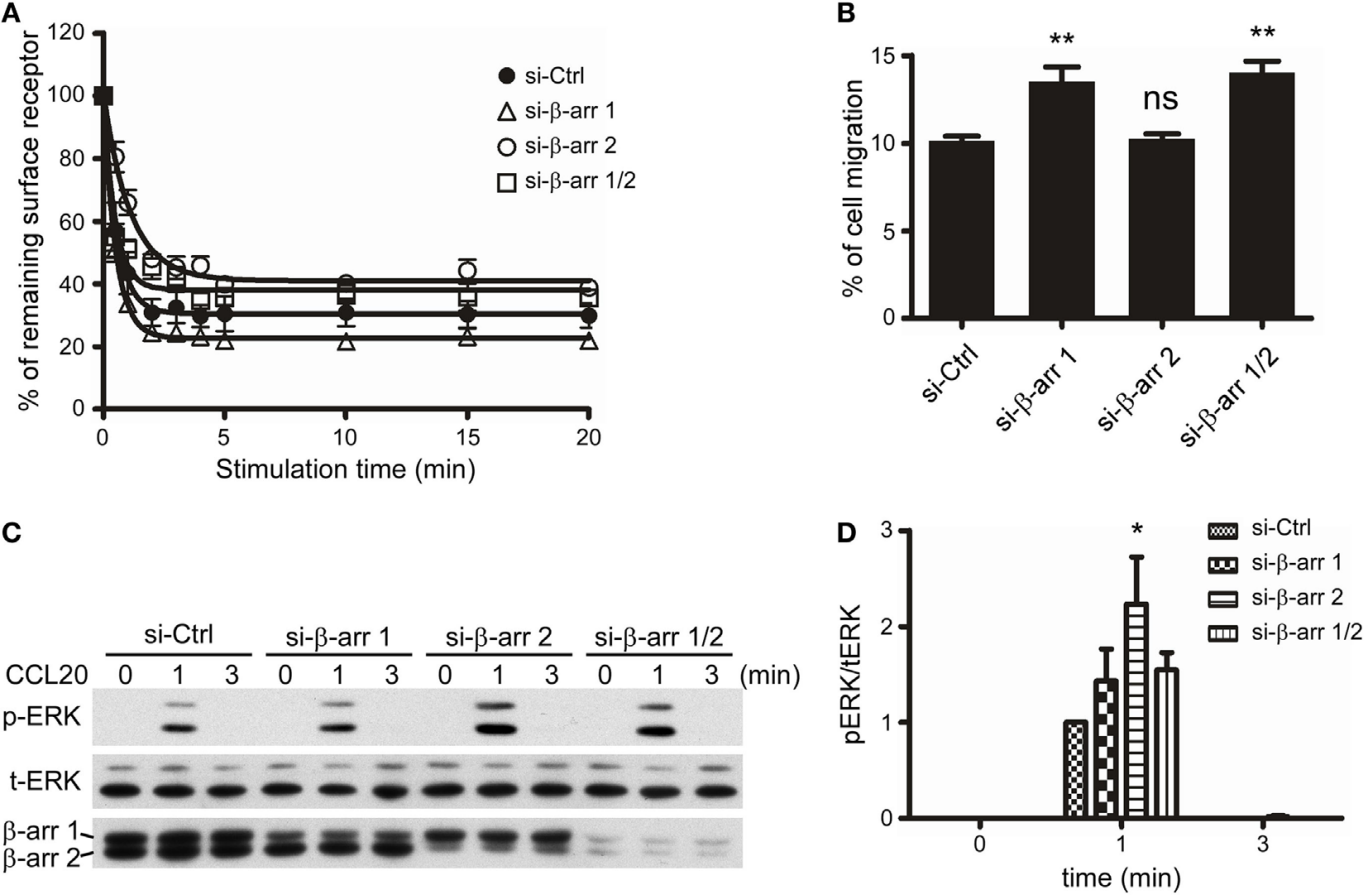
β-Arrestin 1 and β-arrestin 2 play distinct roles in the CCL20-induced CCR6 internalization, cell migration, and ERK1/2 phosphorylation. **(A)** Jurkat cells stably expressing wild-type CCR6 were transfected with non-targeting (Ctrl), β-arrestin 1, or β-arrestin 2 siRNAs for 40 h. Cells were stimulated with CCL20 and analyzed for CCR6 internalization **(A)**, cell migration **(B)**, or ERK phosphorylation **(C)**. **(D)** The ratio of phosphorylated ERK to total ERK was calculated from western blot quantification. Data represent the mean ± SEM from five independent experiments (ns, not significant, *p < 0.05 and **p < 0.01 vs. si-Ctrl).

Since we had demonstrated that C-terminal Ser/Thr phosphorylation impacts CCR6 internalization, chemotaxis, and ERK1/2 activation (Figures 2–7), and that β-arrestin 1 and β-arrestin 2 were able to associate with CCR6 (Figure 8), we next examined the role of endogenous β-arrestin 1 and β-arrestin 2 in CCR6-mediated events. We used siRNA to knockdown endogenous β-arrestin 1 and/or β-arrestin 2 and then examined CCL20-induced receptor internalization, chemotaxis, and ERK1/2 activation. FACS analysis showed that knockdown of β-arrestin 1 promoted CCR6 internalization, whereas knockdown of β-arrestin 2 attenuated CCR6 internalization (Figure 9A). Knockdown of β-arrestin 1 enhanced CCR6-mediated cell migration, whereas knockdown of β-arrestin 2 had no effect on cell migration (Figure 9B). Altogether, these results suggest that β-arrestin 1 and β-arrestin 2 play different roles in CCR6-mediated internalization and cell migration.

Given that β-arrestins function as scaffolds for facilitating GPCR-mediated MAPK activation (23), we also examined the role of β-arrestin 1 or β-arrestin 2 on CCR6-mediated ERK1/2 activation. Our data showed that knockdown of β-arrestin 2 significantly increased CCL20-induced ERK1/2 activation, while knockdown of β-arrestin 1 slightly increased CCL20-induced ERK1/2 activation (Figures 9C,D). Therefore, we conclude that β-arrestin 2 negatively regulates CCR6-mediated ERK1/2 activation. Interestingly, knockdown of both β-arrestin 1 and β-arrestin 2 produced a similar pattern of ERK1/2 activation as knockdown of β-arrestin 1 alone. These results may account for our earlier observation that ERK1/2 activation was reduced compared with WT-CCR6 in 4A-CCR6 transfectants after CCL20 stimulation. This effect would be predicted based on promotion of β-arrestin 2 binding with receptors (Figures 8B–D). Taken together, these results revealed that both β-arrestin 1 and β-arrestin 2 are able to bind phosphorylated CCR6; however, β-arrestin 1 seems to play a regulatory role in CCR6-mediated receptor internalization and cell migration, while β-arrestin 2 seems to play a regulatory role in internalization and transient ERK1/2 activation.

## Discussion

**Figure 10:**
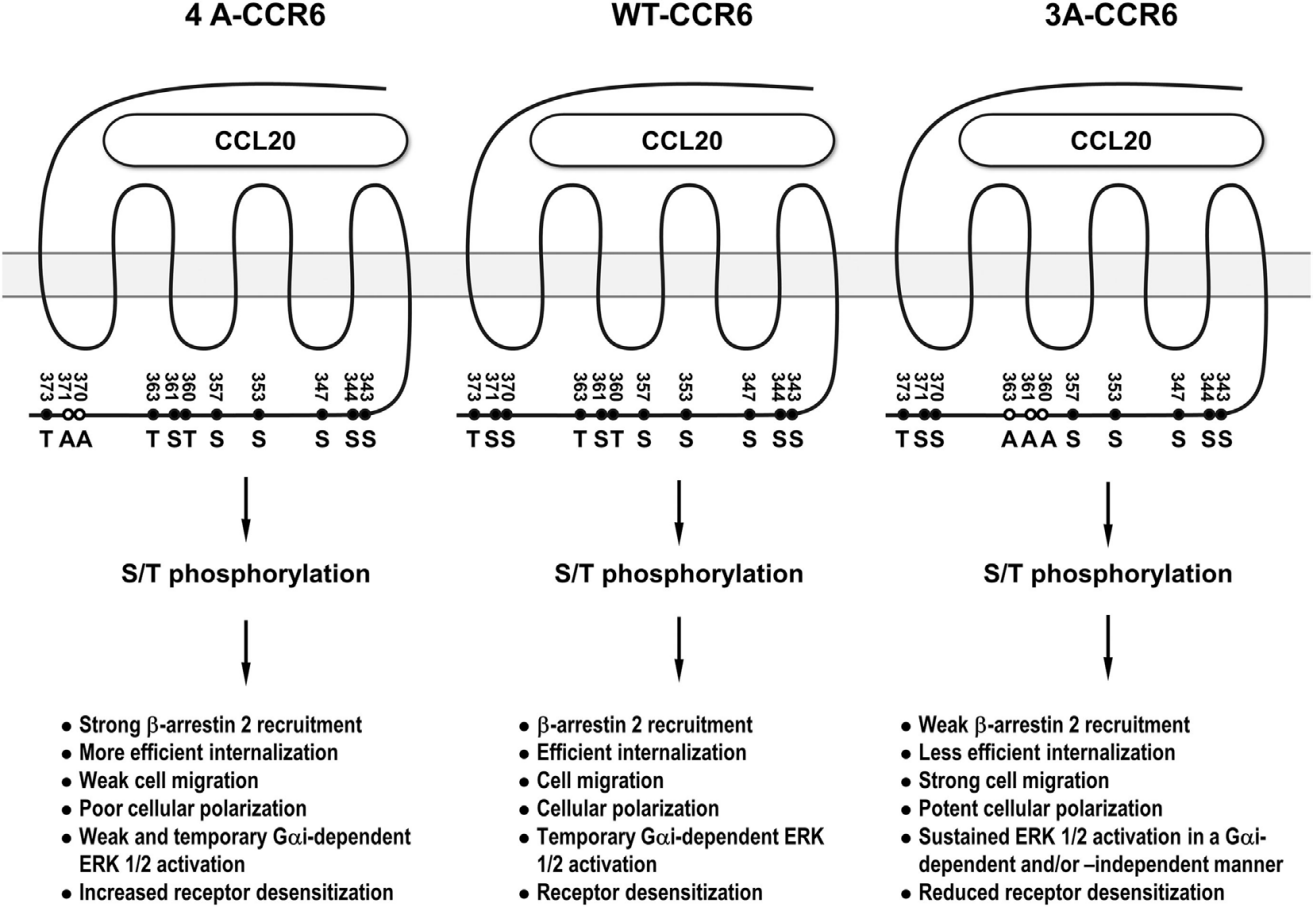
The summary of the differential biological effects mediated by distinct phosphorylation of Ser/Thr residues on the C-terminal tail of CCR6. 3A-CCR6 (Thr360, Ser361, and Thr363 were mutated into Ala) and 4A-CCR6 (Ser370 and Ser371 were mutated into Ala) differentially regulate CCR6-mediated biological effects.

CCR6 is a signature chemokine receptor for Th17 cells (5–8), which have been shown to be associated with many inflammatory diseases (6, 11, 12). Elucidating the underlying signaling that controls the biological function of CCL20/CCR6 may provide important insights that aid in the development of novel therapeutic approaches for Th17-mediated inflammatory diseases. In this study, we demonstrated that distinct clusters of Ser/Thr residues in the CCR6 C-terminus differentially regulate the biological functions of the receptor. We further demonstrated that β-arrestin 1 and β-arrestin 2 also differentially regulate CCR6-mediated functions. Through our mutagenesis experiments, we identified a stretch of C-terminal Ser/Thr residues in cluster 3 (Thr360, Ser361, and Thr363) as being important for internalization, desensitization, and transient ERK activation. We also identified a stretch of C-terminal Ser/Thr residues in cluster 4 (Ser370 and Ser371) as being critical for ERK activation, chemotaxis, F-actin distribution. In addition, we found that β-arrestin 2 associates with the CCR6 C-terminal tail through cluster 3 Ser/Thr residues to positively regulate receptor internalization and negatively regulates transient ERK1/2 activation, while β-arrestin 1 seems to weakly associate with CCR6 and may be involved in negatively regulating receptor internalization and CCR6-mediated cell migration. In sum, differential biological effects mediated by distinct phosphorylation of Ser/Thr residues on the C-terminal tail of CCR6 are shown in Figure 10.

Interestingly, mutating Ser/Thr residues in cluster 3 (Thr360, Ser361, and Thr363) and cluster 4 (Ser370 and Ser371) produced opposing effects. Compared with WT-CCR6, ligand-stimulated 4A-CCR6 exhibited enhanced receptor internalization and desensitization, and decreased chemotaxis, actin polymerization and ERK1/2 activation. By contrast, CCL20-stimulated 3A-CCR6 exhibited decreased internalization and desensitization with increased effects on chemotaxis, actin polymerization and sustained activation of ERK1/2 (Figures 2, 4, 5B,C, 6A, 7 and 8B). These two Ser/Thr clusters within the C-terminal domain of CCR6 may comprise a regulatory code that is capable of positively and negatively regulating CCR6 biological functions. It has been reported that different GRKs may phosphorylate distinct Ser/Thr residues in the C-terminal tail of GPCRs, significantly affecting the topology and charge of the intracellular face of receptors. These physical changes, in turn, may then determine the conformation of recruited β-arrestins and trigger specific, but disparate, downstream signaling (25, 26, 45). It is plausible that Ser/Thr residues in cluster 3 and cluster 4 of the CCR6 C-terminus may be phosphorylated by different GRKs, corresponding to the different biological functions of each cluster. In addition, a very recent study has provided an example where different GPCR-β-arrestin conformations mediate distinct functional outputs (46). Thus, the phosphorylated Ser/Thr residues in cluster 3 and cluster 4 may similarly alter the structure of CCR6, which may then adopt functionally relevant conformations within CCR6-β-arrestin complexes, and thereby produce distinct biological outputs.

Phosphorylation of the C-terminal domain and intracellular loops in GPCRs is required for β-arrestin recruitment that drives receptor internalization and desensitization (22, 47). Based on the stability of interactions between GPCRs and β-arrestins, GPCRs are divided into two classes (48). Class A GPCRs, such as β2-adrenergic receptor (β2AR), transiently interact with β-arrestins and tend to undergo rapid recycling after stimulation. By contrast, class B GPCRs, such as angiotensin receptor subtype 1a (AT1aR), stably interact with β-arrestins and tend to exhibit slow recycling/endosomal sorting (48, 49). In our immunoprecipitation and immunofluorescence experiments, we found that activated CCR6 is able to form complexes with β-arrestin 2-GFP, and complex formation was observed to peak around 5 min, with a rapid decline up to 20 min (Figure 8). Based on the transient nature of this binding profile, CCR6 can be classified as a class A GPCR. Interestingly, the activated mutant, 4A-CCR6, shows an enhanced and prolonged interaction with β-arrestin 2-GFP, with almost completely absent recycling to the cell surface (Figure 3). Thus, the 4A-CCR6 mutant should be considered as a class B GPCR. It is somewhat striking that the inability to phosphorylate two serine residues (Ser 370 and Ser 371) at the CCR6 C-terminus could switch the receptor type from class A to class B. Given that cluster 3 is required for β-arrestin 2 binding and that cluster 3 phosphorylation in the absence of cluster 4 phosphorylation results in a stabilized interaction with β-arrestin 2, it is plausible that phosphorylation of cluster 4 may play a role in preventing excess phosphorylation of cluster 3. This type of phosphorylation tempering may be a key factor which makes CCR6 a class A GPCR.

G protein-coupled receptor-induced ERK activation can be Gαi-dependent and/or β-arrestin dependent (50), and the duration and intensity of ERK1/2 activation are crucial determinants in the cellular response to GPCR activation (51). β-Arrestin association with the C-terminal tail of GPCRs has been shown to positively regulate ERK activation (50). As such, class B GPCRs, such as AT1aR, strongly bind β-arrestins and exhibit prolonged ERK activation (52, 53), whereas class A GPCRs, such as β2AR, transiently bind β-arrestins and exhibit less persistent β-arrestin-mediated ERK activation. Surprisingly, activated 4A-CCR6 shows stable and prolonged association with β-arrestin 2, but it does not show sustained ERK1/2 activation. Instead, the ligand-stimulated ERK1/2 activation level was lower than WT-CCR6 (Figures 6A and 8B). By contrast, activated 3A-CCR6 weakly associates with β-arrestin 2, but it stimulates enhanced and sustained ERK1/2 activation (Figures 6A and 8B). This set of observations is of special interest because very few reports have shown that β-arrestin binding negatively correlates with G protein-dependent ERK1/2 activation (54). However, GPCR-mediated ERK activation is known involve complex molecular signaling. In fact, GPCR stimulation of ERK activation may reflect heterogeneous signaling events that are receptor and cell-type dependent, resulting in fine spatial and temporal control of the ERK signal (55). For example, a recent study has shown that the muscarinic receptor, M_1_R, regulates ERK activation by two modes of arrestin binding. First, β-arrestin provides a scaffold and activates ERK when it stably binds to phosphorylated M_1_R. By contrast, however, transient binding of β-arrestin reduces ERK activation *via* the recruitment of a protein phosphatase (56). Since we observed that CCL20/CCR6-induced ERK activation is transient (Figures 6, 7 and 9), we speculate that CCR6-induced ERK activation may be initially Gαi-dependent. Subsequently, the activated ERK may bind β-arrestin scaffolds, which also recruit protein phosphatase to diminish ERK activity. This potential explanation would account for our dual observations that 4A-CCR6 shows the strongest interaction with β-arrestins, but has the weakest ERK activation (Figure 6), and that β-arrestin 2 knockdown results in increased ERK1/2 activation (Figure 9).

Although β-arrestin 1 and β-arrestin 2 show high sequence and structural homology, they have strikingly differential functionalities (45, 54, 57). In our study, we also observed that β-arrestin 1 and β-arrestin 2 play distinct roles in CCR6-mediated functions (Figure 9). Knockdown of β-arrestin 2 results in reduced CCR6 internalization (Figure 9A), suggesting that β-arrestin 2 is critical for this process. The mutagenesis experiments showed that activated 3A-CCR6 failed to interact with β-arrestin 2 (Figure 8B). It is plausible that CCR6 activation induces phosphorylation of Ser/Thr residues in cluster 3, allowing the binding of β-arrestin-2, and subsequently driving CCR6 internalization (Figure 2) and desensitization (Figure 7). On the other hand, our data show that β-arrestin 1 weakly interacts with activated CCR6 (Figure 8A) and that this arrestin interacts equally with all CCR6 mutants. Thus, the specific binding motif that allows CCR6 to bind β-arrestin 1 is still unidentified (Figure 8A; Figure S4 in Supplementary Material). Whether the weak association of β-arrestin 1 with activated CCR6 may interfere with the recruitment of β-arrestin 2 is an open question that requires further investigation.

The major function of chemokine receptor signaling is to stimulate leukocyte migration. Rearrangement of the actin cytoskeleton is an early cellular event that is required for chemokine-induced cell polarization and cell migration. Moreover, coordination of sequential actin assembly and disassembly is required to drive directional cell migration (42). Our data showed that CCL20 stimulation induces a rapid accumulation of intracellular F-actin that is different in magnitude and kinetics between WT and various mutant CCR6-expressing cells (Figure 5). Stimulation of 3A-CCR6, 4A-CCR6, and 34A-CCR6 alters actin rearrangement, suggesting that distinct C-terminal Ser/Thr phosphorylation events regulate actin polymerization, depolymerization, and chemotaxis. Peculiarly, if the phosphorylation of CCR6 C-terminal Ser/Thr residues regulates actin polymerization and chemotaxis, it may be predicted that knockdown of β-arrestins would impair actin polymerization and cell migration as well. However, knockdown of β-arrestin 2 had no effect on cell migration and knockdown of β-arrestin 1 increased cell migration. Thus, the question of how distinct C-terminal Ser/Thr phosphorylation events regulate cell migration will require further study.

Our study provides novel insight into how clusters of C-terminal Ser/Thr residues in CCR6 are involved in regulating CCL20-induced receptor internalization, cellular chemotaxis and ERK1/2 activation. In line with the barcode hypothesis that has been previously advanced (28, 39), our findings indicate that site-specific phosphorylation of Ser/Thr residues in CCR6 can elicit different signaling outcomes. These barcode-directed outcomes may be mediated by β-arrestins and/or other proteins. Indeed, we demonstrated that β-arrestin 1 and β-arrestin 2 play coordinated but distinct roles in mediating cellular responses to CCR6 activation.

In general, similar mechanisms govern the signal transduction of all chemokine receptors except atypical chemokine receptors; however, chemokines may activate distinct signaling pathways in various contexts, such as in different cell types. A single chemokine may bind to multiple receptors on distinct cells to induce different signaling and biological function. Furthermore, cell-specific signaling machinery and individual ligands may confer differential affinity and/or structural motifs for receptor binding to downstream effector molecules, leading to distinct biological outcomes. For example, the two cognate ligands for CCR7 (CCL19 and CCL21) induce differential signaling pathways in dendritic cells (31). Stimulation of receptors for CXCL8, CXCR1, and CXCR2, also give rise to distinct functional outcomes in RBL-2H3 cells (36). For CXCR4, Ser/Thr phosphorylation has been reported on the C-terminal domain (33, 34) or/and on intracellular loops 2 or 3 (34) to transduce CXCL12-mediated biological effects in HEK293 cells. In this study, we show that Ser/Thr phosphorylation of CCR6 occurs on the C-terminal domain. Given that chemokines and chemokine receptors are involved in many inflammatory diseases, chemokine receptors have been widely investigated as therapeutic agents. Our study and others demonstrate that for potential drug-targeting application, specific signaling outcomes for each chemokine receptor should be carefully investigated in the context of target cell types. By doing so, investigators may develop promising druggable target molecules for inflammatory diseases.

## Author Contributions

M-YL designed and performed experiments, analyzed data, and wrote the manuscript. S-SL performed experiments. S-LC performed experiments. FL supervised and designed experiments, analyzed data, and wrote the manuscript.

## Conflict of Interest Statement

The authors declare that the research was conducted in the absence of any commercial or financial relationships that could be construed as a potential conflict of interest.
