# Supplementary material for: The Phosphorylation of CCR6 on Distinct Ser/Thr Residues in the Carboxyl Terminus Differentially Regulates Biological Function
Source: Front Immunol. 2018 Mar 2;9:415. doi: 10.3389/fimmu.2018.00415 (PMC5840145; doi:10.3389/fimmu.2018.00415)
Supplement: Supplementary file 1 [file Data_Sheet_1.PDF]

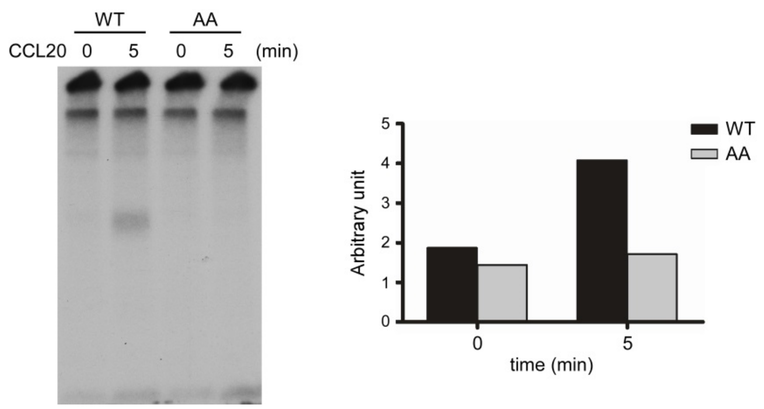

Supplementary Figure 1. CCR6 phosphorylation was readily detected in WT-CCR6 but not AA-CCR6 transfectants in response to CCL20 stimulation. (A) HEK293T cells were transfected with HA-tagged WT-CCR6 or AA-CCR6 constructs for 24 h and then fasted in phosphate-free DMEM medium containing 0.5% BSA and 10 mM HEPES for 3 h at 37 °C. Cells were then trypsinized and resuspended in phosphate-free DMEM at a density of 1×10^7 cells/ml. Next, cells were metabolically labeled with 60 μCi/ml of P^{32} for 1.5 h at 37°C followed by the stimulation with 100 ng/ml CCL20 for 0 or 5 min. Finally, cells were subjected to immunoprecipitation with HA-tagged CCR6 using anti-HA affinity gel. The immunoprecipitates were run on 9% SDS-PAGE and the signals were detected and quantified by the Typhoon 9410 imaging system (B).

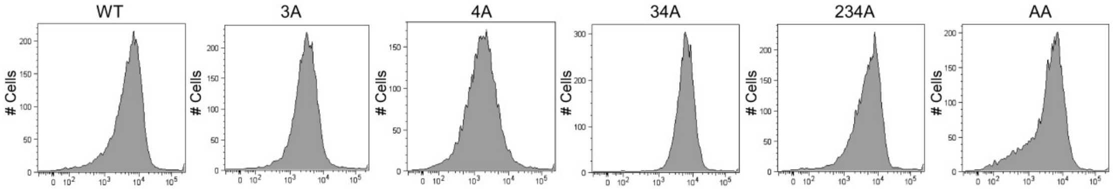

Supplementary Figure 2. Surface levels of CCR6 in transfectants. Jurkat cells stably expressing HA-tagged WT-CCR6, 3A-CCR6, 4A-CCR6, 34A-CCR6, 234A-CCR6, or AA-CCR6 showed similar CCR6 surface levels by flow cytometry analysis.

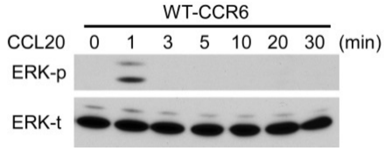

Supplementary Figure 3. CCL20/CCR6 signaling induces transient ERK phosphorylation. Jurkat cells that stably express WT-CCR6 were fasted and then stimulated with 100 ng/ml CCL20 for 0, 1, 3, 5, 10, 20 or 30 min. Cell lysates were analyzed by immunoblotting using anti-phospho-ERK1/2 (ERK-p) and anti-total ERK (tERK).

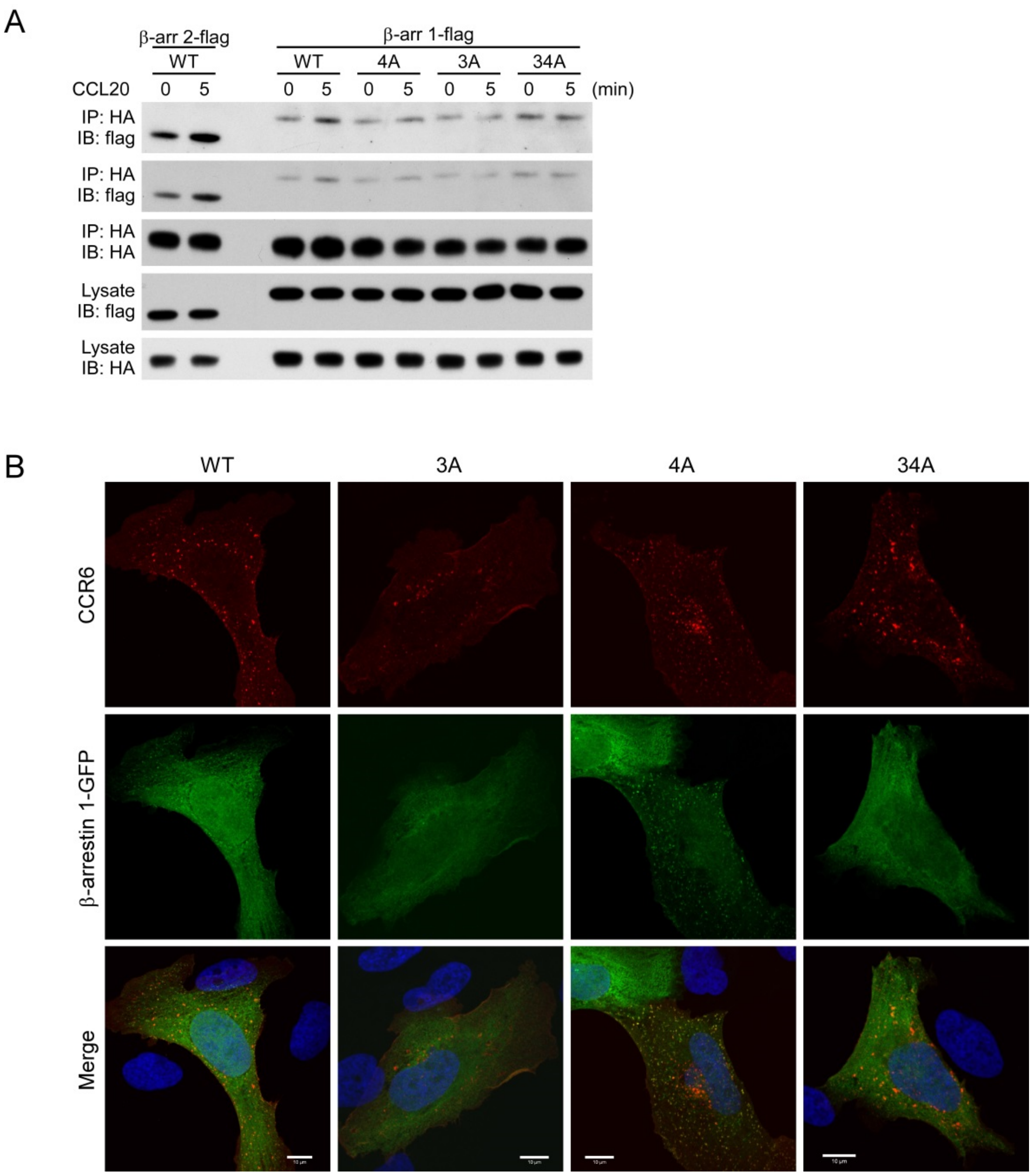

Supplementary Figure 4. HEK293T cells co-transfected with β-arrestin 1-flag or β-arrestin 2-flag and HA-WT-CCR6 or various CCR6 mutant constructs for 24 h were trypsinized and stimulated with 100 ng/ml CCL20 for 0 or 5 min. Cell lysates were immunoprecipitated with anti-HA agarose followed by western blotting using anti-flag and anti-HA antibodies. The transfected β-arrestin 1-flag, β-arrestin 2-flag and HA-WT-CCR6 in whole cell lysates were detected using anti-flag and HA antibodies. (B) U2OS cells co-transfected with β-arrestin 1-GFP (green) and WT-CCR6, 3A-CCR6, 4A-CCR6 or 34A-CCR6 constructs for 24 h were surface stained with anti-CCR6 antibody followed by stimulation with 100 ng/ml CCL20 for 5 min. Cells were subsequently fixed and stained with anti-mouse-Alexa 594 (red) and DAPI (blue). Images were obtained with a LSM700 confocal microscope. Scale bar = 10 μm.

Supplementary Table 1

Primers for site-directed mutagenesis	Sequence (5' -> 3')	Template/generated construct
S347A-F	GTCCTCAGGCTTCGCCTGTGCCGGGAGGTAC	234A/1234A
S347-R	GTACCTCCCGGCACAGGCGAAGCCTGAGGAC	
S343A/S344A-F	GTGAGAAGGAAGTACAAGGCCGCAGGCTTCGCCTGTGCC	
S343A/S344A-R	GGCACAGGCGAAGCCTGCGGCCTTGTACTTCCTTCTCAC	
S353A/S357A-F	CGGGAGGTAC GCA GAAAACATT GCT CGGCAGACCAG	34A/234A
S353A/S357A-R	CTGGTCTGCCG AGC AATGTTTTC TGC GTACCTCCCG	
T360A/S361A/T363A-F	CATTGCTCGGCAG GCC GCT GAG GCC GCAGATAACGAC	WT/3A, 4A/34A
T360A/S361A/T363A-R	GTCGTTATCTGC GGC CTC AGC GGC CTGCCGAGCAATG	
S370A/S371A-F	GCAGATAACGACAATGCG GCG GCC TTCACTATGTG	WT/4A
S370A/S371A-R	CACATAGTGAA GGC CGC CGCATTGTCGTTATCTGC	
T373A-F	CGGCGGCCTTC GCT ATGTGATAGAAAGCTG	1234A/AA
T373A-R	CAGCTTTCTATCACAT AGC GAAGGCCGCCG	

Primer for	Sequence (5' ->3')	construct
Rat β-arrestin 1-HindIII-F	CCCAAGCTTCGGACCATGGGCGACAAAGGGACA	Rat β-arrestin 1-GFP
Rat β-arrestin 1-PstI-R	TGCACTGCAGTCTGTTGTTGAGGTGTGGAGA	
Human β-arrestin 2-HindIII-F	CCCAAGCTTCGCACCATGGGGGAGAAACCC	β-arrestin 2-GFP or b-arrestin 2-flag
Human β-arrestin 2-flag-EcoRI-F	GGAATTCCTACTTGTCGTCATCGTCTTTGTAGTCGCAGAG TTGATCATCATAGTC	β-arrestin 2-flag
Human β-arrestin 2-flag-EcoRI-R	GGAATTCCCTTGTCGTCATCGTCTTTGTAGTCGCAGAGTT GATCATCATAGTC	β-arrestin 2-GFP
